# Development of a glass-based imaging phantom to model the optical properties of human tissue

**DOI:** 10.1364/BOE.504774

**Published:** 2023-12-21

**Authors:** Mingze Yang, Yunle Wei, Philipp Reineck, Heike Ebendorff-Heidepriem, Jiawen Li, Robert A. McLaughlin

**Affiliations:** 1 School of Biomedicine, The University of Adelaide, Adelaide, SA, Australia; 2 Institute for Photonics and Advanced Sensing, The University of Adelaide, Adelaide, SA, Australia; 3 School of Physics, Chemistry and Earth Sciences, The University of Adelaide, Adelaide, SA, Australia; 4 School of Science, RMIT University, Melbourne, VIC, Australia; 5 School of Electrical and Mechanical Engineering, The University of Adelaide, Adelaide, SA, Australia

## Abstract

The fabrication of a stable, reproducible optical imaging phantom is critical to the assessment and optimization of optical imaging systems. We demonstrate the use of an alternative material, glass, for the development of tissue-mimicking phantoms. The glass matrix was doped with nickel ions to approximate the absorption of hemoglobin. Scattering levels representative of human tissue were induced in the glass matrix through controlled crystallization at elevated temperatures. We show that this type of glass is a viable material for creating tissue-mimicking optical phantoms by providing controlled levels of scattering and absorption with excellent optical homogeneity, long-term stability and reproducibility.

## 1. Introduction

Optical imaging modalities are becoming increasingly significant for their ability to measure the interaction of light with biological tissues to assess disease [1,2]. Examples include diffuse optical imaging [3], Raman spectroscopy [4] and fluorescence imaging [5]. In designing and developing these imaging modalities, consensus standards are required for performance evaluation, routine calibration and cross-comparison between imaging systems. This avoids the implicit variation that arises over time from using different samples of *in vivo* or *ex vivo* tissue. These standards must exhibit reproducible, homogeneous and long-term stable optical properties [1,6,7], in order to measure the reliability of imaging systems [2]. To provide an indication of system performance with tissue imaging, these optical properties are developed to be similar to those of biological tissue. Such standards are referred to as tissue-mimicking optical phantoms. As tissues present with a wide range of optical properties, various types of tissue-mimicking phantoms have been proposed for different optical imaging modalities [2].

In many designs of tissue-mimicking optical phantoms, two key properties, optical scattering and absorption, have been included by incorporating absorption and scattering agents in a transparent matrix material to achieve controllable optical properties [1,2,4,8]. Categorized by the matrix material, tissue-mimicking optical phantoms can be divided into water, hydrogel, and non-water-based polymer phantoms. Water-based phantoms have advantages with their low-cost and simple fabrication procedures. However, the liquid phase of water-based phantoms precludes the possibility of incorporating complex structures in the phantoms, such as inclusions with different optical properties or multiple layers [1,2,9]. An alternative type of matrix material is hydrogel, which is composed of a cross-linked network such as agar, gelatin and polyvinyl acetate (PVA) that retains water [1,2]. The network allows hydrogels to be structured into different shapes. For example, hydrogel-based phantoms have been shaped into organ phantoms with similar mechanical properties to real human tissues [10] or more complex geometrical structures for imaging system calibration purposes [11]. However, if inappropriately stored, they will suffer from short-term stability due to the high water content in the phantom matrix. A hydrogel-based phantom may only last from a few hours exposed to air to several months in hydrated conditions [1,4,8]. Improved long-term stability has been demonstrated through the use of a non-water-based polymer as a matrix material [2]. Examples include polyester and polyurethane resin [12], silicone elastomer polydimethylsiloxane (PDMS) [9] and polyvinyl chloride (PVC) [13,14].

In order to mimic absorption in human tissues, absorption agents have been selected to target specific tissue types or clinical applications. Natural absorption agents, including whole blood [15], hemoglobin dye [16], melanin [14] and some food color dye [17], have been used in both water- and hydrogel-based phantoms. These phantoms can present a similar absorption spectrum to real human tissues, but the stability of these absorption agents may only last for a few days [1,2]. Other absorption agents, such as India Ink [18] and molecular dyes [19], have also been investigated in many phantom designs based on water, hydrogel and polymer as they can provide a broad or peaked absorption spectrum with better long-term stability compared to biological absorption agents [1,2].

Optical scattering can be imitated in a phantom using scattering agents with different refractive indexes to the matrix materials [2,20]. One approach to induce scattering is adding ex-situ made scattering agents to the phantom matrix. For example, lipid emulsion is a common ex-situ scattering agent used for water- and hydrogel-based phantoms, which provides consistent optical properties over different batches with a low absorption coefficient [1]. Other ex-situ scattering agents, such as silica beads [21] and polymer microspheres [22], have been used in hydrogel- and non-water polymer-based phantoms [1]. Both silica beads and polymer microspheres exhibit well-controlled particle size and refractive index. This allows the prediction of scattering levels of tissue-mimicking phantoms based on Mie scattering theory as a function of the refractive index and size of the spherical particles [1,2]. Another type of ex-situ scattering agents are white metal oxide powders, such as titanium dioxide (TiO_2_) [23], aluminium oxide (Al_2_O_3_) [24] and zinc oxide (ZnO), which have been used for hydrogel- and non-water-based polymer phantoms. These metal oxide particles have a high refractive index providing high levels of optical scattering while exhibiting low absorption, and have been widely used for the development of tissue-mimicking phantoms [1,8,9].

A different approach to induce scattering in a matrix is the in-situ formation of scattering agents. An example is the freezing-thawing process for PVA-based phantoms [25]. With increased cycles of the freezing-thawing process, the polymer network within the matrix is enhanced, which results in an increasing level of optical scattering in the PVA phantoms. However, the repeatability of this method is limited because small variations in the fabrication procedure can impact the scattering properties of the PVA phantoms [2]. Another example is to mix glycerol in PDMS, which creates spherical air-filled cavities with low refractive index in the matrix [9]. Based on the refractive index difference between air and PDMS matrix, the scattering levels can be controlled by varying the concentration of glycerol in the phantom design.

In this paper, we present an alternative material for the development of tissue-mimicking phantoms. Inorganic glass is chosen as the matrix material as it offers excellent long-term stability combined with the ability to induce absorption and scattering in the glass matrix. We fabricated glass samples with varying absorption and scattering levels to show the potential of using glass as a matrix material for human tissue-mimicking phantoms. Absorption is induced by doping the glass with nickel ions that mimic hemoglobin absorption. High and low levels of absorption are achieved by controlling the concentration of nickel ions doped into the glass matrix. Scattering is achieved by in-situ formation of crystals in the glass upon heat treatment, which provides a homogenous distribution of scattering species in the glass matrix. By applying different temperatures and durations of heat treatment, the size of the crystals is varied, enabling control of the level of scattering. The optical properties of the glass samples were evaluated over the wavelength range of 400 nm – 800 nm. In order to assess the reliability of glass as a matrix material for phantom applications, the homogeneity, reproducibility and long-term stability of samples were tested. The high homogeneity, reproducibility and long-term stability of the glass samples demonstrate their excellent suitability for use in tissue-mimicking phantoms.

## 2. Glass-based phantom design

This section describes the design of the glass, with the aim of identifying the most suitable materials and fabrication method to achieve tissue-like levels of absorption and scattering. The fabrication of the glass matrices is based on the melt-quenching technique [26], which requires melting a batch of crystalline raw glassmaking materials at an elevated temperature. For the fabrication of the glass samples in this work, a melting temperature of 1400 °C was used. By contrast, the previously reported water-, hydrogel- and polymer-based phantoms are commonly made at lower temperatures (below 200 °C), so hereafter those phantoms are referred to as low-temperature phantoms to distinguish them from glass-based phantoms made at high temperature.

Typical absorption agents used for the fabrication of low-temperature phantoms are organic compounds, such as whole blood, Indian Ink, molecular dye and carbon powders, which degrade at temperatures above 400 °C. These materials cannot be used to fabricate high-temperature glass-based phantoms. Therefore, alternative approaches are required for inducing absorption in glass-based phantoms. The absorption of glass in the visible spectrum is commonly achieved by doping metal ions or forming metal or semiconductor nanoparticles in the glass matrix [27,28]. In this work, we aim to mimic the absorption of hemoglobin which has an absorption peak at a wavelength of ∼435 nm. For this purpose, nickel ions, silver nanoparticles and ZnSe semiconductor nanoparticles are suitable absorption agents as their absorption spectra are similar to that of hemoglobin. In contrast to the metal or semiconductor nanoparticles, inducing controlled levels of absorption from nickel ions is readily achieved by making the glass from a batch of raw glass making materials containing a prescribed amount of nickel oxide. Therefore, we selected nickel ion doping for inducing absorption for the first demonstration of the potential of glass as matrix material for phantom applications.

Similar to incorporating absorption agents in glass-based phantoms, the scattering agents that can be used for glass-based phantoms are limited by the high glass melting temperature. For example, it is difficult to adopt the method of incorporating white metal oxide particles (e.g. TiO_2_), that is used in low-temperature phantoms, for the fabrication of glass-based phantoms as these metal oxide particles are easily dissolved in a glass melt [26]. Therefore, different methods for incorporating scattering agents are required for the fabrication of glass-based phantoms. Using the glass-ceramics method [27,29], crystals are formed by heating the glass in bulk form first at a base temperature, where a large number of crystal seeds are formed, and then at a slightly higher temperature to grow these seeds into crystals, from a few nanometers to hundreds of nanometers in diameter. For this approach, the level of scattering in glass depends on the number, size and size distribution of the crystals. These features can be readily controlled by the heating temperatures and times. Thus, we used the in-situ formation of crystals to induce scattering in the glass for use in phantom applications.

## 3. Experiment

### 3.1. Glass sample selection and fabrication

The fabrication of a series of glass samples that are appropriate for use in glass-based phantoms was modified from an existing dense opal glass-ceramics patent (US2559805A) [30]. All glass samples were fabricated using the composition of 59SiO_2_-18Na_2_O-16BaO-7P_2_O_5 _+ xNiO (in weight %), where x = 0.25 and 0.5 corresponding to the low and high levels of optical absorption from nickel ions. High-purity reagents of SiO_2_ (99.995%, Advalue Technology), Na_2_CO_3_ (99.997%, Thermo Fisher), BaCO_3_ (99%, Sigma Aldrich) and (NH_4_)_3_PO_4_ (99.99%, Sigma Aldrich) were used as the raw glassmaking materials.

**Fig. 1.**
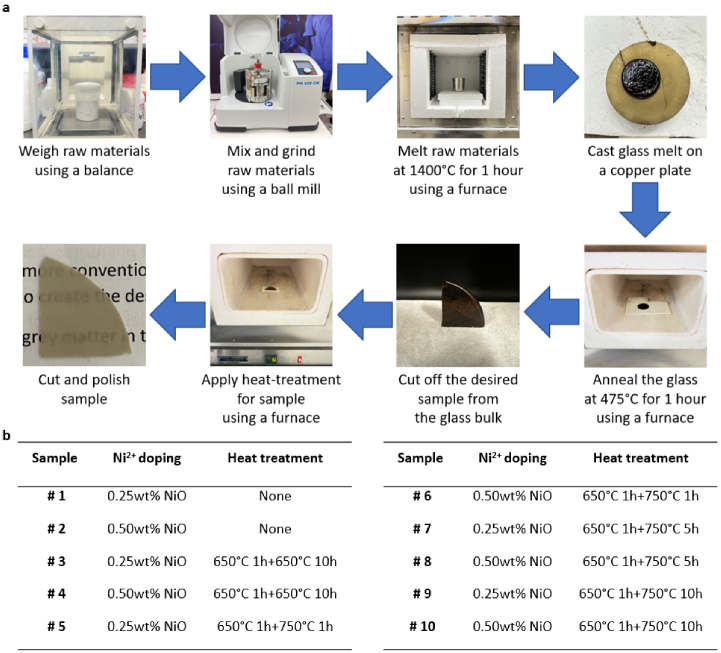
(a) Flow chart of the fabrication of the glass samples. (b) List of glass samples with different amount of NiO doping and varying temperature and time of heat treatment.

As shown in Fig. 1(a), the raw glassmaking materials were first weighed by a laboratory balance. Then, the raw materials were mixed and finely ground using a ball mill (Planetary Ball Mill PM 100 CM, Retsch, Germany). The mixed materials were then heated up in an electrical resistance laboratory furnace (customized furnace made by Tetlow Kilns & Furnaces, Australia) to 1400 °C in a platinum crucible and maintained for 1 hour to completely melt the raw materials and homogenize the glass melt. The melt was cast onto a copper plate and immediately pressed with a second copper plate in order to accelerate the cooling rate and avoid crystallization at this stage. The cast glass was immediately annealed at 475 °C for 1 hour and allowed to gradually cool down to room temperature to release internal stresses. Glass samples were round-shaped with a diameter of 8 cm and thickness of 4 mm, and were subsequent cut into smaller samples as required as shown in Fig. 1. At this stage, the samples were often stored in the laboratory at room temperature for a period of one to two days. There was no variation observed in the characteristics of the glass samples over these periods. The glass samples were observed to show excellent transparency with minimal scattering. A homogeneous brown colour was observed in the samples due to the homogeneous dispersion of the nickel ions throughout the glass matrix. These glass samples were used to demonstrate the ability to control the absorption levels in the glasses.

We induced scattering through in-situ formation of crystals in the glass matrix upon heat treatment. Based on the study of crystallization in soda lime glass and silicophosphates glass [31–33], we assumed the temperature for the nucleation and crystal growth to be around 650 °C and 750 °C, respectively. Thus, to induce different levels of optical scattering in the glass matrix, the glass samples were first heated at 650 °C for 1 hour in an electrical resistance laboratory furnace (customized furnace made by Tetlow Kilns & Furnaces, Australia) to create crystal seeds in the glass matrix, and then further heated at 650 °C or 750 °C to grow these seeds into large crystals. This 2-stage heat-treatment induced crystal sizes that resulted in an opaque appearance. The final stage of the fabrication procedure involved sample surface polishing that finished with a 0.5 µm cerium oxide polishing suspension. These glass samples (Samples #3 – #10 listed in Fig. 1(b)) were used to demonstrate the ability of controlling scattering in the glass.

### 3.2. Characterization of the crystals formed in the glass samples

The crystals in the glass samples were visualized by taking Backscattered Electron (BSE) images using an Ultra-High-Resolution Scanning Electron Microscope (SEM) (SU7000, Hitachi, Japan). For each of the glass sample (Samples #4, #6, #8 and #10), a small piece was cut off from the bulk sample and polished on one side and carbon-coated. In order to visualize the crystals in the glass matrix, the SEM was operated at 3 kV. The BSE images were taken at the center of each glass sample. Then, the histogram of each sample was plotted to present the size distribution of the crystals in the sample matrix.

After imaging, the piece of Sample #10 was cleaned with ethanol and ground into powder, which was used to measure X-Ray diffraction (XRD) with an X-Ray Diffractometer (MiniFlex 600, Rigaku, Japan). The type of crystals formed in the samples after heat treatment was determined by comparing the diffraction peak position of the powder under test to the XRD standard cards.

### 3.3. Measurement of the optical properties of the samples

**Fig. 2.**
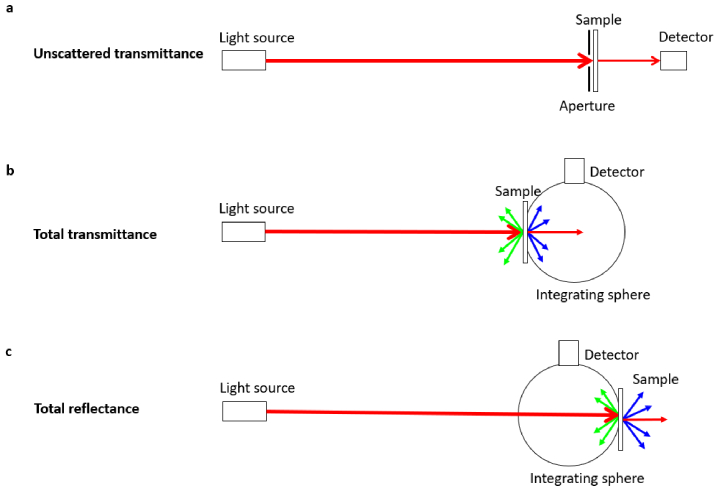
Schematic of the set-ups with/without an external integrating sphere for the measurement of (a) unscattered transmittance, (b) total transmittance and (c) total reflectance.

The optical properties (i.e. unscattered transmittance *U*, total transmittance *T* and total reflectance *R*) of the samples were measured using a dual-beam UV-Vis spectrophotometer (Cary 5000, Agilent Technologies, USA) in two configurations. The unscattered transmittance was measured without an integrating sphere with an aperture of 5 mm diameter placed in front of the sample (Fig. 2(a)). For the measurement of the total transmittance and reflectance, an external integrating sphere (DRA-1800, Agilent Technologies, USA) was connected to the spectrophotometer and used with a 5 mm small spot kit and a 99% white standard reference (Spectralon, Edmund Optics, USA) as described in detail by Prahl [34]. For the total transmittance measurement, the sample was positioned at the integration sphere entrance port with the white standard located at the exit port (Fig. 2(b)), enabling the total transmittance to be measured relative to no sample located at the entrance port. For the reflectance measurement, the sample was located at the exit port of the integrating sphere with a slight angle (Fig. 2(c)). This configuration allowed the specular reflection to be included in the measurement of the total reflectance relative to the white standard.

As Samples #1 and #2 were not subjected to heat treatment for in-situ crystal formation, they showed negligible scattering. Hence, the attenuation in the volume is only caused by the absorption in the glass sample, which can be calculated from the unscattered transmittance as $A={\text{log}}_{10}(1/U)$ . The sample was polished on both sides to a thickness for the measurement of the unscattered transmittance. The absorption coefficient *µ_a0_* in the absence of scattering was calculated from the attenuation *A* and the sample thickness *d* by

(1) $$\mu_{a0}=\frac{A-A_{\text{min}}}{d}*ln10$$

where the attenuation at each measured wavelength was subtracted by the minimal attenuation value around 750 nm (where nickel ions do not present absorption) to eliminate the attenuation from surface effects such as Fresnel reflectance and surface defects from polishing.

As Samples #3 – #10 were subjected to heat treatment, they presented both absorption and scattering. We found that the use of a small sample thickness of 180 µm allowed sufficient light passing through for unscattered and total transmittance measurements. The inverse adding doubling (IAD) algorithm [35] was used to determine the absorption coefficient *µ_a_* and reduced scattering coefficient *µ_s_’* from the total reflectance, total transmittance, and unscattered transmittance spectra.

### 3.4. Assessment of the reliability of the samples

Reliability of the glass samples was evaluated by individually assessing the homogeneity, reproducibility and long-term stability of the glass samples. These reliability properties are affected by the variation of scattering and absorption for the samples, which changes the attenuation within or between the samples. Thus, we measured the attenuation to analyze the reliability of the samples, and quantified this by calculating the coefficient of variation (CV), which is defined here as the ratio of the standard deviation to the mean value of the attenuation [36].

For the assessment of the optical homogeneity, four samples with high and low levels of scattering and absorption (Samples #5, #6, #9 and #10) were selected. Their unscattered transmittance was measured at 5 locations (four at the edges of the sample and one at the center of the sample) across each of the four samples.

For the assessment of optical reproducibility, Sample #10 with high scattering and absorption was selected as the representative sample for variation in optical properties. The fabrication of Sample #10 was repeated five times over different days. All five Samples #10a – #10e were made following the same fabrication procedure for achieving high scattering and high absorption. The unscattered transmittance was measured at the center of each of the five samples.

For the assessment of the long-term stability, four samples exhibiting the combinations of two levels of scattering and absorption (Samples #5, #6, #9 and #10) were selected. In order to assess the optical reliability over a long time, we performed an accelerated aging test following the accelerated aging test protocol described by Krauter et al. [37] of placing the samples in an oven at 60 °C. The test was conducted over 28 days to simulate a one-year storage condition with an ambient temperature of 25 °C. At Day 0 (prior to heating) and at each subsequent seven days, the unscattered transmittance was measured at the center of each sample.

## 4. Results and discussion

### 4.1. Absorption in the glass samples

**Fig. 3.**
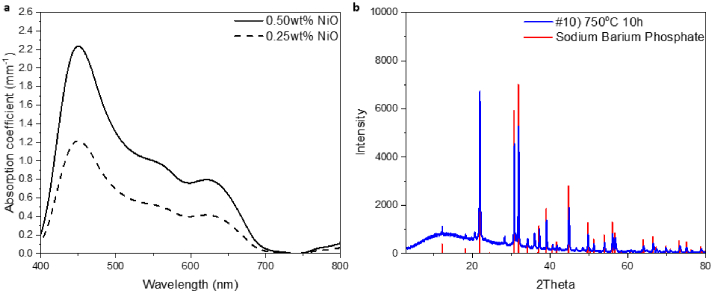
(a) Absorption coefficient *µ_a0_* of the nickel-doped glass Samples #1 and #2 made without heat treatment. (b) XRD plot of the Sample #10 (made using heat-treatment at 750 °C for 10 hours) (blue line) and XRD standard card of Sodium Barium Phosphate crystal (red line).

Figure 3(a) shows the absorption coefficient *µ_a0_* in the absence of scattering in the spectral range from 400 nm – 800 nm for Samples #1 and #2, which were made without heat treatment and hence without inducing in-situ crystallization. The spectra exhibit an absorption band with peak wavelength around 425 nm, which presents a comparable peak position to that of the absorption band of hemoglobin. Compared to Sample #1 with lower nickel oxide content of 0.25 wt%, Sample #2 with two times higher nickel oxide content of 0.50 wt% has an almost two times higher absorption coefficient at 450 nm (1.21 mm^−1^ for Sample #1 and 2.24 mm^−1^ for Sample #2) and an exactly two times higher absorption coefficient at 621 nm (0.40 mm^−1^ for Sample #1 and 0.80 mm^−1^ for Sample #2). The absorption is approximately proportional to the concentration of nickel ions in the batch of glassmaking raw materials. In agreement with the Beer-Lambert law, where the absorption is proportional to the concentration of absorbing ions in the glass matrix, this indicates that the concentration of nickel oxide in the glass batch leads to the intended concentration of nickel ions in the glass matrix. Therefore, the absorption level of the glass sample can be controlled by varying the amount of nickel oxide in the raw material.

### 4.2. Scattering in the glass samples

As shown in Fig. 3(b), the XRD pattern of Sample #10, which was made using heat-treatment of 650 °C for 1 hour followed by 750 °C for 10 hours (blue line), presents comparable peaks to the standard data (JCPDS No: 81-2250) (red line), indicating the scattering species formed in the glass matrix were sodium barium phosphate (NaBaPO_4_) crystals.

**Fig. 4.**
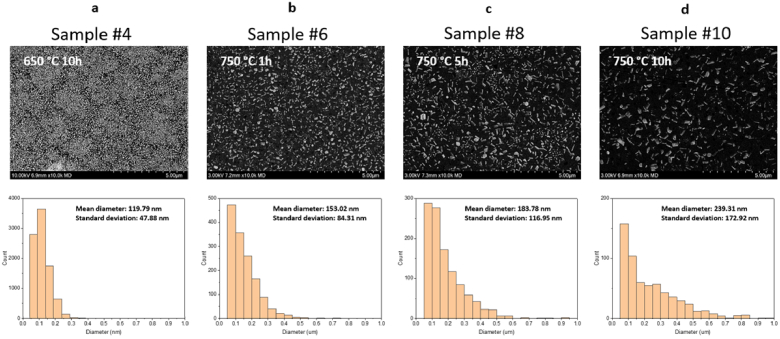
(Top) BSE images of the Samples #4, #6, #8 and #10 made using different heat-treatments with (bottom) corresponding histogram of crystal diameter.

The BSE images of the samples made using heat-treatment at 750 °C show that the crystals have irregular shape (Fig. 4(a) – (d)). Therefore, Feret’s diameter, i.e. the longest distance between two points on the boundary of each crystal particle, is used to quantify the size of the crystals. The average size of the crystals in each sample is defined as the mean value of the Feret’s diameter of the measured sample. The histograms of the crystal size distribution for the BSE images show that the average crystal size is significantly enlarged when the sample was subjected to the heat-treatment at 750 °C in comparison to the sample treated at 650 °C for 10 hours. As shown in Fig, 4, even after applying a long duration of heat-treatment at 650 °C in the nucleation temperature range, only small crystals were formed in the glass matrices. Therefore, if larger crystal sizes are required to induce higher scattering, a second heat-treatment at 750 °C is necessary for the crystal growth.

Considering the samples made using heat treatment at 750 °C, the average crystal size was found to increase with increasing duration of the heat treatment (from 153 nm for 1-hour heat-treatment to 239 nm for 10-hour heat-treatment), whereas the number of distinct crystals in the glass matrix shown in the image decreases with increasing duration of the heat-treatment. By determining pixel counts of crystals for each image (Fig. 4(b) – (d)), we found that the pixel counts of crystals in the glass matrices were comparable for all the glass samples treated at 750 °C despite different heat treatment durations. This indicates that the crystal phase portion did not change with longer duration of the heat treatment at 750 °C. As the crystals in the glass matrix were identified as NaBaPO_4_ with molar composition 3.5Na_2_O-7BaO-3.5P_2_O_5_, and the molar composition of glass samples is 69SiO_2_-20Na_2_O-7BaO-4P_2_O_5_, the amount of BaO and P_2_O_5_ is considered to limit the growth of the crystals in the glass matrix. We hypothesize all the BaO and P_2_O_5_ in the glass matrix were already incorporated in the crystals after 1-hour heat treatment. Thus, no further Ba^2+^ and P^5+^ were available in the glass matrix for crystal growth at longer heat-treatment durations. Nevertheless, the formation of larger crystals with longer heat-treatment durations was observed, but with a reduced number density of crystals in the matrix. This behavior is consistent with Oswald ripening mechanism for crystal growth, where large crystals grow via dissolution of small crystals, which maintains the total amount of crystalline phase [31,33].

**Fig. 5.**
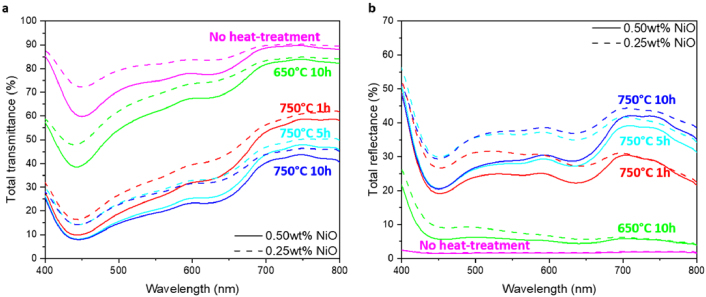
(a)Total transmittance and (b) total reflectance of the Samples #1 – #10. The solid and dash lines correspond to the 0.50wt% and 0.25wt% of nickel ions in the matrix, respectively.

The total transmittance and reflectance spectra of the samples made without the heat treatment, and with different temperatures and durations for the second heat treatment are shown in Fig. 5(a) and (b). Both the scattering of the crystals and the absorption of the nickel ions in the glass matrices affect the transmittance and reflectance in the glass samples in the range of 400 nm – 700 nm. In order to investigate the effect of the crystals on the scattering levels in the glass samples, we compared the transmittance and reflectance spectra at 750 nm, where nickel ions present neglectable absorption. Compared to Samples #1 and #2 made without heat treatment, the Samples #3 and #4 made using second heat treatment at 650 °C for 10 hours show slightly lower total transmittance and slightly higher total reflectance, whereas Samples #5 – #10 made using second heat-treatment at a higher temperature of 750 °C show significantly lower transmittance and higher reflectance at 750 nm. This indicates that high level of scattering was induced in the glass by the higher temperature for the second heat treatment.

**Fig. 6.**
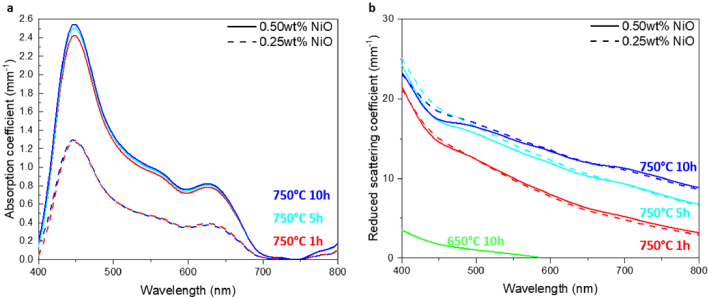
(a) Absorption coefficient *µ_a_* and (b) reduced scattering coefficient *µ_s_’* of the Samples #5 – #10. The solid and dash lines correspond to the 0.50wt% and 0.25wt% of nickel ions in the glass matrix, respectively.

The absorption coefficient spectra and reduced scattering coefficient spectra determined using the IAD algorithm for the samples made with different durations for the second heat treatment at 750°C are shown in Fig. 6(a) and (b). In addition, the reduced scattering coefficient of the sample made with a 10-hour heat treatment at 650 °C is shown. The latter sample presents a low value of the reduced scattering coefficient of ∼0.71 mm^−1^ at 532 nm (green curve in Fig. 6(b)). This result is consistent with the observation of a large number of small crystals in this sample (Fig. 4(a)), indicating that even long heat treatment at 650 °C only leads to the formation of small crystals that cause low level of scattering, which is insufficient to mimic many types of human tissues. This result confirms that a second heat treatment at 750 °C is necessary to enlarge the crystal size and, in this way, to increase the scattering level in the glass matrix to sufficient level for the fabrication of glass-based phantoms.

The low scattering of the samples made with a 650 °C second heat treatment present very low levels of reflectance when using the same sample thickness as other samples made with 750 °C second heat treatment, which prevented reliable determination of the absorption coefficient with the IAD algorithm. Thus, the absorption coefficients are only discussed for the samples made with 750 °C second heat treatment. Comparing the absorption coefficients of the samples with low nickel ion doping level (Samples #5, #7, #9) and high nickel ion doping level (Samples #6, #8, #10) (Fig. 6(a)), doubling of the nickel oxide content from 0.25 wt% to 0.50 wt% almost doubled the peak absorption coefficient at 450 nm from around 1.3 mm^−1^ to around 2.5 mm^−1^. This result is consistent with Beer-Lambert law, which matches the relationship between absorption level and nickel oxide content in the samples made without scattering as described in Section 4.1.

The samples doped with the same amount of nickel oxide in the glassmaking material have almost identical absorption coefficient spectra (Fig. 6(a)). For both low and high nickel ion content, the samples made with heat treatment at 750 °C have similar shape compared to that of the samples made without heat treatment as shown in Supplement 1, Fig. S1. These results indicate that the absorption of the glass samples is not affected by the in-situ formation of the crystals in the glass matrix.

The reduced scattering coefficient at 400 nm – 800 nm increases with increasing duration of the second heat treatment at 750 °C (Fig. 6(b)). This behaviour correlates with increasing average crystal size from 153 nm to 239 nm for prolonged heat treatment (Fig. 4(b) – (d)) but not with the slightly decreasing number density of the crystals in the glass matrix. This correlation indicates that the average crystal size has the dominating effect on the reduced scattering coefficient, which agrees with the simulation of Mie scattering for small particles with particle size below 1 µm as shown in the Supplement 1 Fig. S2 [38]. For the same heat-treatment temperature and time, the samples with low and high nickel ion content exhibit similar reduced scattering coefficients (Fig. 6(b)). The samples with higher nickel ion content show slightly lower scattering coefficient around 450 nm compared to the samples with lower nickel ion content. In this wavelength range, the unscattered transmittance is very low as shown in the Supplement 1 Fig. S3, which prevents reliable determination of reduced scattering coefficient with the IAD algorithm. The similarity of the reduced scattering coefficient spectra (Fig. 6(b)) suggests that the nickel-ion doping does not affect the scattering, i.e. the in-situ formation of the crystals in the glass matrix. Therefore, the reduced scattering coefficient can be independently controlled by varying the parameters of the second heat-treatment, regardless of the amount of nickel-ion doping in the glass matrix.

### 4.3. Reliability assessment of the glass samples

**Fig. 7.**
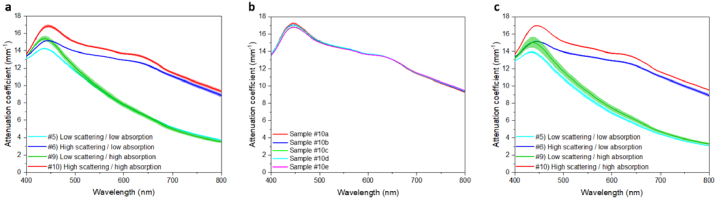
(a) Mean attenuation of Samples #5, #6, #9, #10, each measured at five different locations (all combinations of low and high scattering and absorption), the error bars are the standard deviation of the attenuation measured at different locations. Error bars are shown as shaded areas around each plot. (b) The attenuation spectra of Samples #10a-#10e fabricated using the same procedure. (c) Mean attenuation of Samples #5, #6, #9, #10, measured over one-month accelerated aging, the error bars are the standard deviation of the attenuation measured at different days.

Samples #5, #6, #9 and #10 were selected for the assessment of the homogeneity, reproducibility and long-term stability, as these samples show both absorption and scattering suitable for the design of tissue-mimicking phantoms. The four samples exhibit different combinations of high and low absorption and scattering. Figure 7 shows the average value of the measured attenuation, where the error bars show the standard deviation of the measured attenuation.

The homogeneity of the glass samples was demonstrated by measuring the attenuation at five different locations on each of four glass samples. The error bars of the attenuation spectra are negligible (Fig. 7(a)), demonstrating a high level of homogeneity of both scattering and absorption (CV value below 2% over the entire measurement range in the visible spectral region).

Reproducibility was assessed by measuring the attenuation on five samples (#10a – #10e) with the same material composition and fabrication process. The attenuation spectra of these samples are comparable (Fig. 7(b)), indicating that the samples can be fabricated with a high degree of inter-batch reproducibility in absorption and scattering levels (CV value below 2%).

The long-term stability of the glass samples was examined by measuring the attenuation on each of four samples during the accelerated-aging experiment. The samples subjected to the 4-week accelerated-aging experiment show a low standard deviation in their attenuation spectra (Fig. 7(c)), indicating that the glass samples maintain stable scattering and absorption levels over one-year-equivalent room-temperature conditions (CV value below 4%). The slightly higher variance in Sample #9 arose primarily from a single measurement (Week 0) and may be due to slight inconsistencies in the measurement process rather than changes in the sample.

## 5. Conclusion and outlook

We have utilized inorganic glass as a novel matrix material that is suitable for use in optical phantoms to mimic the optical properties of human tissues. Nickel ions doped into the glass were used to induce absorption similar to that of hemoglobin. The absorption level of the nickel ions was controlled by varying the content of nickel oxide in the raw material mixture from which the glass was made. Crystals formed in-situ in the glass upon heat treatment were used to induce scattering similar to that of the human tissues. The average size of the crystals in the glass matrix increased with extended durations of heat treatment, while the total crystal phase portion was retained. This behavior aligns with Oswald ripening mechanism of crystal growth and allows control of the scattering level through heat treatment duration. As the presence of nickel ions does not affect the crystal formation in the glass matrix and the crystals do not induce absorption in the matrix, the levels of absorption and scattering can be independently controlled, highlighting the viability of this glass as a phantom matrix material suitable for mimicking a range of tissue types. The glass samples showed excellent optical stability, while also demonstrating excellent optical homogeneity, reproducibility and long-term stability. In practice, we noted that the glass samples were robust and remained intact without any instances of cracking or breaking during measurements and tests under normal handling condition.

This work represents an initial proof-of-concept study, which shows limitations and scope for improvement. For applications requiring an accurate mimicking of the hemoglobin spectrum, silver [28] and gold nanoparticles [39] are promising absorption agents, as they present an absorption peak at around 410 nm and 550 nm, respectively. The absorption spectrum of these nanoparticle doped glasses is affected by both the concentration and the morphology of the nanoparticles. These factors can be tuned by adjusting the quantity of silver or gold ions in the glassmaking materials and by controlling the conditions during the glass heat-treatment process [28]. By modifying the nanoparticle size, it is possible to shift the absorption peak. This may allow mimicking of other tissue chromophores, such as bilirubin. However, challenges remain to simultaneously match both the spectral position and width of the absorption peak over a wide range of wavelengths.

A further limitation lies in the irregular shape of the in-situ crystals formed in the glass matrix. For applications requiring specific shapes of scattering agents, different morphology and composition of the in-situ grown crystal phase may be achieved by using different glass compositions and heat-treatment. For example, lithium disilicate glass ceramics demonstrate the capability of forming spherical Li_3_PO_4_ crystals in the glass matrix [33].

Prior studies have established that different types of human tissue present varying levels of reduced scattering coefficient. For instance, white matter can have a reduced scattering coefficient exceeding 10 mm^−1^ around 500 nm and approximately 9 mm^−1^ around 800 nm [40], whereas subcutaneous fat shows reduced scattering coefficient of 2.5 mm^−1^ at 500 nm and 2 mm^−1^ at 800 nm [41,42]. In comparison, the reduced scattering coefficients of the glass samples heat treated at 750 °C exceed the highest scattering level of human tissues. In contrast, the sample heat treated at lower temperature of 650 °C exhibits a lower reduced scattering coefficient which is in the scattering range of human tissues. This indicates that the methodology of utilizing in-situ formed crystals to induce scattering in a glass-based phantom matrix material can be adapted, e.g. by modifying the heat treatment temperature, to adjust the reduced scattering coefficients for mimicking a specific type of human tissue. It is worth noting that the size of the scattering crystals formed in the glass matrix are smaller than the wavelengths of light used in this paper for the characterization of the samples. The small crystal size led to Rayleigh scattering rather than Mie scattering which is more prevalent in tissue [43,44]. For optical applications which require scattering agents greater than 1 µm, alternative glass compositions, such as Li_2_O-CaO-SiO_2_ glass-forming system [45], can be explored to facilitate the growth of larger crystals within the glass matrix.

Sodium leaching has been observed, particularly in humid environments, which is attributed to the high sodium content in the glass matrix. Thus, low humidity conditions would be required to maximize the stable shelf-life of the glass. We also note that the refractive index of our glass is around 1.53, which is higher than most tissue. These limitations can be mitigated by using a glass composition that exhibits a lower refractive index and lower sodium ion content. Tailoring of the glass composition as well as absorption and scattering agents in glass allows extension of the optical properties of glass into the UV and/or NIR range, which could enable the development of glass-based phantoms over a broad range.

The glass samples described in this work may be fabricated at a larger scale or with more intricate shapes through the use of customized molds for casting. In addition, the use of the emerging technology of 3D printing of fused silica glass [46,47] enables the possibility of development of 3D printed glass-based phantoms with complex and high-resolution structures. This could allow for the incorporation of specific optical properties, such as absorption [46], scattering [48], fluorescence [49], etc. Such improvements in the development of glass-based phantoms could extend flexibility and precision in mimicking optical properties of biological tissues for various optical systems.

## Data Availability

Data underlying the results presented in this paper are not publicly available at this time but may be obtained from the authors upon reasonable request.

## Supplemental document

See Supplement 1 for supporting content.
