# Supplementary material for: Development of a glass-based imaging phantom to model the optical properties of human tissue
Source: Biomed Opt Express. 2023 Dec 21;15(1):346–59. doi: 10.1364/BOE.504774 (PMC10783914; doi:10.1364/BOE.504774)
Supplement: Supplementary file 1 [file boe-15-1-346-s001.pdf]

Supplemental Document

Biomedical Optics EXPRESS

Development of a glass-based imaging phantom to model the optical properties of human tissue: supplement

MINGZE YANG,[1,2] YUNLE WEI,[2,3] PHILIPP REINECK,[4] HEIKE EBENDORFF-HEIDEPRIEM,[2,3] JIAWEN LI,[2,5] AND ROBERT A. MCLAUGHLIN[1,2,*]

[1] *School of Biomedicine, The University of Adelaide, Adelaide, SA, Australia*
[2] *Institute for Photonics and Advanced Sensing, The University of Adelaide, Adelaide, SA, Australia*
[3] *School of Physics, Chemistry and Earth Sciences, The University of Adelaide, Adelaide, SA, Australia*
[4] *School of Science, RMIT University, Melbourne, VIC, Australia*
[5] *School of Electrical and Mechanical Engineering, The University of Adelaide, Adelaide, SA, Australia*
[*] *robert.mclaughlin@adelaide.edu.au*

This supplement published with Optica Publishing Group on 21 December 2023 by The Authors under the terms of the Creative Commons Attribution 4.0 License in the format provided by the authors and unedited. Further distribution of this work must maintain attribution to the author(s) and the published article's title, journal citation, and DOI.

Supplement DOI: https://doi.org/10.6084/m9.figshare.24720225

Parent Article DOI: https://doi.org/10.1364/BOE.504774

Development of a glass-based imaging phantom to model the optical properties of human tissue: Supplementary Information

MINGZE YANG,[1,2] YUNLE WEI,[2,3] PHILIPP REINECK,[4] HEIKE EBENDORFF-HEIDEPRIEM,[2,3] JIAWEN LI,[2,5] AND ROBERT A. MCLAUGHLIN[1,2,*]

[1]*School of Biomedicine, The University of Adelaide, Adelaide, SA, Australia.*
[2]*Institute for Photonics and Advanced Sensing, The University of Adelaide, Adelaide, SA, Australia.*
[3]*School of Physics, Chemistry and Earth Sciences, The University of Adelaide, Adelaide, SA, Australia.*
[4]*School of Science, RMIT University, Melbourne, VIC, Australia.*
[5]*School of Electrical and Mechanical Engineering, The University of Adelaide, Adelaide, SA, Australia.*
**robert.mclaughlin@adelaide.edu.au*

1. Normalized absorption spectra of the glass samples

As the samples without the heat-treatment process (Sample #1 and #2) show minimum scattering in the glass matrices, the inverse adding doubling (IAD) algorithm [1, 2] was unable to calculate their absorption coefficient. Attenuation in these samples only resulted from the absorption of the samples. Therefore, the absorption of these samples was calculated from their unscattered transmittance, as $A = log_{10}(1/U)$. The absorption coefficient of Samples #5 – #10 was obtained by IAD algorithm from the measured total reflectance and total transmittance. In order to compare the shape of the absorption spectra of these samples, the absorption peaks of all the samples were normalized to 1 and the minimum values were normalized to 0 (Fig. S1), eliminating the effects of volume scattering, Fresnel reflectance, surface scattering and thickness.

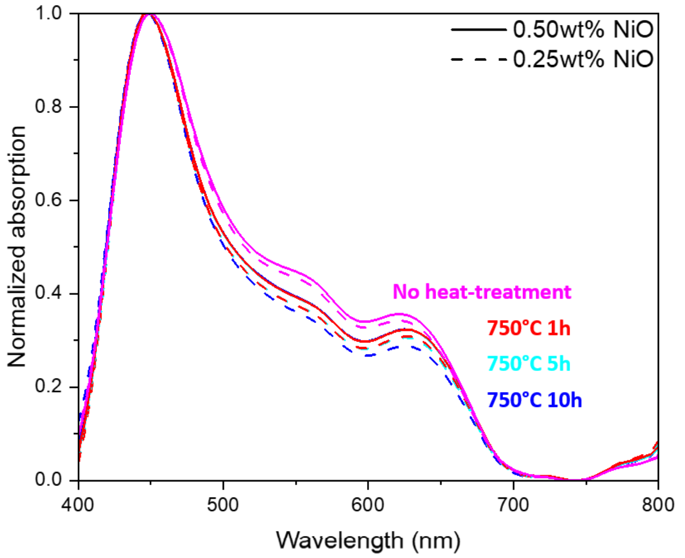

Fig. S1. The normalized absorption spectra of the glass samples with and without heat-treatment.

2. Simulation of reduced scattering coefficient

In order to simulate the relationship between the reduced scattering coefficient and the crystal parameters (i.e. size, size distribution, concentration), MieSimulatorGUI v1.3 (https://virtualphotonics.org/software-mie-simulator) was used in this work. We note that the particles in the simulation are spherical but the crystals formed in the glass matrices in this work are irregular. Therefore, though the particle size, size distribution and

concentration were obtained from the SEM images of the samples, the reduced scattering coefficients from the simulation software were found to be different to the experimentally measured number. The graphs only show the generic trend of reduced scattering coefficient level with the increasing particle sizes and the decreasing number density but do not quantify the reduced scattering coefficient of different samples. The refractive indices of the crystals and glass matrices were assumed as 1.7 and 1.53.

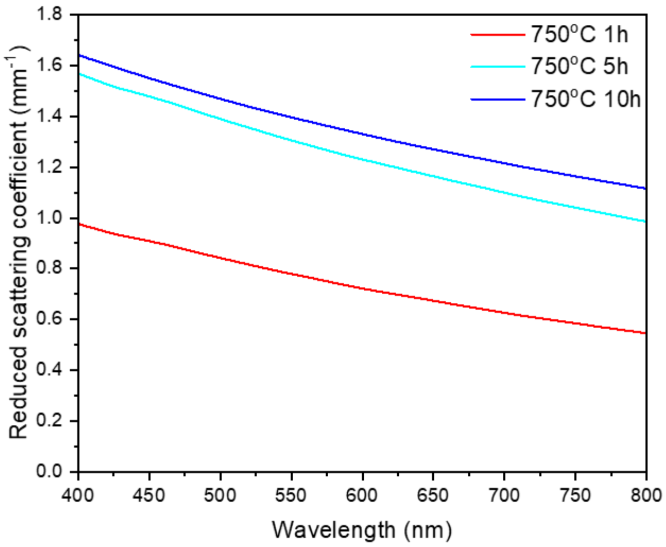

Fig. S2. Simulation of reduced scattering coefficient of the glass samples with increasing crystal diameters and decreasing crystal number density.

3. Unscattered transmittance

Fig. S3 shows the unscattered transmittance of Sample #1 – #10 measured by the UV-Vis spectrophotometer (Cary 5000, Agilent Technologies, USA). The samples were affixed to a sample holder and measured using a 5 mm aperture.

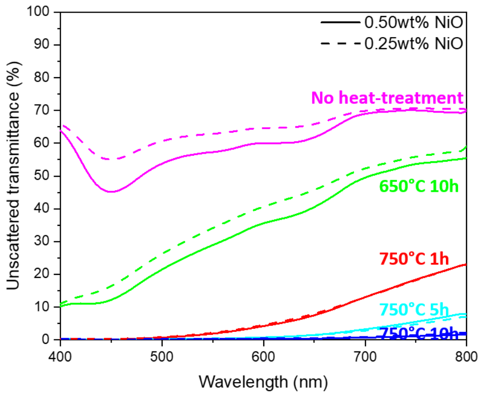

	Ni^{2+} doping	Heat treatment
# 1	0.25wt% NiO	None
# 2	0.50wt% NiO	None
# 3	0.25wt% NiO	650°C 1h+650°C 10h
# 4	0.50wt% NiO	650°C 1h+650°C 10h
# 5	0.25wt% NiO	650°C 1h+750°C 1h
# 6	0.50wt% NiO	650°C 1h+750°C 1h
# 7	0.25wt% NiO	650°C 1h+750°C 5h
# 8	0.50wt% NiO	650°C 1h+750°C 5h
# 9	0.25wt% NiO	650°C 1h+750°C 10h
# 10	0.50wt% NiO	650°C 1h+750°C 10h

Fig. S3. Unscattered transmittance (left) of the Sample #1 – #10 (right). The solid and dash lines correspond to the 0.50wt% and 0.25wt% of nickel ions in the matrix, respectively

References

1. Prahl, S. Everything I think you should know about Inverse Adding-Doubling. March 2011; Available from: https://omlc.org/software/iad/manual.pdf.

2. Prahl, S.A., M.J.C. van Gemert, and A.J. Welch, Determining the optical properties of turbid media by using the adding–doubling method. Applied Optics, 1993. **32**(4): p. 559-568.
